# Prevalence and bacteriology of culture-positive urinary tract infection among pregnant women with suspected urinary tract infection at Mbarara regional referral hospital, South-Western Uganda

**DOI:** 10.1186/s12884-021-03641-8

**Published:** 2021-02-23

**Authors:** Bahati Johnson, Bawakanya Mayanja Stephen, Ngonzi Joseph, Owaraganise Asiphas, Kayondo Musa, Kabanda Taseera

**Affiliations:** 1 Department of Obstetrics and Gynaecology, Mbarara University of Science and Technology, Mbarara City, Uganda; 2 Mbarara Regional Referral Hospital, Mbarara City, Uganda; 3 Department of Microbiology, Mbarara University of Science and Technology, Mbarara City, Uganda

**Keywords:** Urinary tract infection (UTI), Pregnant women, Bacteriology, Multi-drug resistance, Symptomatic UTI

## Abstract

**Background:**

Urinary tract infections (UTIs) in pregnant women contribute about 25% of all infections and are among the most frequent clinical bacterial infections. Pregnancy changes in women that include anatomical, physiological and hormonal make them susceptible to develop UTI. Left untreated, UTI in pregnancy is associated with grave complications to the mother and fetus. These complications can be decreased by prompt and proper diagnosis and appropriate treatment that also reduces the emergency of drug resistance. Antimicrobial resistance is a major health problem in the treatment of UTI. We determined the prevalence, bacteriology and antimicrobial susceptibility of symptomatic urinary tract infection among pregnant women at Mbarara Regional Referral Hospital.

**Methods:**

We conducted a cross-sectional study from November 2019 to February 2020 involving 400 pregnant women with symptomatic UTI. Patient information was obtained using a structured questionnaire. We collected clean-catch midstream urine specimens for culture and performed antimicrobial susceptibility testing following Clinical and Laboratory Standards Institute standards. Data was entered into RED-cap Version 8.2 software and then exported to Stata Version 14.1 for analysis.

**Results:**

The proportion of culture-positive UTI was 140/400 (35%). Gram-negative bacteria were more prevalent (73%): *Klebsiella pneumoniae* 52(37.41%), *Escherichia coli* 40(28.78%), *Pseudomonas aeruginosa* and *Proteus mirabilis* 7(5.04% each), Citrobacter freundii 1(1%). *Staphylococcus aureus* 33(23.57%) was the only gram-positive isolate. All the isolates were resistant to ampicillin, amoxicillin, amoxicillin/clavulanic acid and ceftazidime/clavulanic acid (95.7, 95.0, 72.9 and 50.7% respectively). Prevalence of extended-spectrum beta-lactamases producing Enterobacteriaceae was 29.0% while that of methicillin-resistant *Staphylococcus aureus* was 33.3%. All cultures demonstrated resistance to more than one drug. Majority of the bacterial isolates were sensitive to ciprofloxacin, ceftriaxone, nitrofurantoin, cefotaxime and gentamicin at 82.9, 81.4, 79.3, 78.6, 66.4 and 65.7% respectively.

**Conclusions:**

*Klebsiella pneumoniae* was the most prevalent isolate followed by *E. coli*. These two organisms were highly resistant to the commonly used antibiotics. Our study recorded a higher prevalence of culture-positive UTI in pregnancy than all the studies in Uganda. Empirical treatment of UTI should be minimized as sensitivity varies for each organism, for each drug and over time.

**Supplementary Information:**

The online version contains supplementary material available at 10.1186/s12884-021-03641-8.

## Background

Urinary tract infection (UTI) is an inflammation caused by the presence and growth of microorganisms anywhere in the urinary tract. This could be the lower UTI (urethritis, cystitis) and/or upper UTI (pyelonephritis). The gold standard for the diagnosis of a urinary tract infection is the detection of the pathogen in urine in the presence of clinical symptoms particularly in patients with non-specific symptoms. The pathogen is detected and identified by urine culture which also allows quantitative estimation of bacteriuria. The minimum level of bacteriuria demonstrating an infection of the urinary tract has been defined to be a count of 10^3^ CFU/mL with significant pyuria [1, 2].

Urinary tract infections in pregnant women contribute about 25% of all infections and are among the most frequent clinical bacterial infections. Pregnancy changes in women that include anatomical, physiological and hormonal make them susceptible to develop UTI. The changes include dilatation of urethra, increased bladder volume and decreased bladder tone, decreased ureteral tone that leads to increased urinary stasis and vesico-ureteric reflux. These changes are partly due to increased levels of progesterone and estrogen but also due to pressure created by the growing uterus [3]. Up to 70% of women during pregnancy develop glycosuria, which encourages bacterial growth in the urine [4]. Untreated UTI in pregnancy is associated with complications like pyelonephritis, sepsis, severe sepsis and septic shock, hypertensive disease of pregnancy, anaemia, acute and chronic renal failure, intrauterine growth restriction, premature delivery, foetal mortality, and increased caesarean delivery [3]. These complications can be decreased by proper and prompt diagnosis and treatment of UTI in pregnancy [5].

Management of UTI at Mbarara regional referral hospital (MRRH) has been largely empirical without the use of a urine culture and susceptibility testing to guide therapy. This practice is a risk for development of antimicrobial resistance among uropathogens. Else-where, antimicrobial resistance is a major health problem in the treatment of UTI caused by *Escherichia coli* and Klebsiella pneumonia, the dominant uropathogens in pregnant women [3, 6–9]. At Mulago National Referral Hospital, the largest Hospital in Uganda, it was found that 96% of pregnant women with UTI were treated empirically with 18% having extended spectrum ß-lactamases (ESBL) and 36% with multidrug resistant *Escherichia coli* strains [10]. Therefore, there was a need to identify which bacterial strains are associated with symptomatic UTI in pregnancy at MRRH and document their susceptibility to commonly utilized antimicrobials in order to guide treatment and prevent the emergency of multi- and extremely drug resistant bacterial strains, as well as reduce expenditure on the patient and hospital associated with the treatment of these resistant bacterial strains. This study sought to determine the prevalence of symptomatic UTI, identify the bacteria and susceptibility to selected antimicrobial agents in pregnant women at Mbarara regional referral hospital.

## Methodology

### Study design, study site, study population and selection criteria

This was a cross-sectional study that was carried out among pregnant women with suspected UTI at an outpatient’s clinic and antenatal ward of Mbarara Regional Referral Hospital, South Western Uganda from November 2019 to February 2020. We included all pregnant women with suspected UTI in pregnancy based on the symptoms including lower abdominal pain, frequency of micturition, burning micturition, painful micturition, nausea and/or vomiting, hematuria, and fever, Participants provided written consent. We excluded pregnant women who failed to produce urine and those with vaginal bleeding.

### Study procedure

The sample size estimation was done using Kish Leslie (1965) formula. The minimum number of respondents was calculated to be 400 whom we selected using consecutive sampling. All pregnant women at the Antenatal Clinic and at the Antenatal ward of MRRH with suspected UTI in pregnancy were selected for the study. Each patient who provided written informed consent was administered a questionnaire by the study team. The questionnaire used in our study was developed for this study (attached as supplementary material). The pregnant women were interviewed using pre-coded, pretested, interviewer administered questionnaires to collect patients’ socio-demographic characteristics, medical history including symptoms of UTI, current and past history of antibiotic use and chronic medical conditions. The study team explained to the patient how to collect a clean-catch MSU sample. A toilet with a sink, soap and running water was used. Each participant was counseled to first wash hands with soap and water before proceeding to collect urine while in the toilet. While squatting on the toilet, participants were told to swab the vulva 3 times, from front to backwards using pre-packed sterile pieces of gauze swabs soaked in saline. Emphasis was put on vulva swabbing from front to back and not back to front. With one hand a woman was advised to separate the labia and allowed the first drops of urine into the toilet. Using a big mouth sterile bottle already labeled with her identification, each participant collected a urine sample of at least 30 ml and immediately covered and sealed the bottle and handed it over to study team. All specimens were transported using a cold box to the Microbiology laboratory within a maximum of thirty minutes after collection for processing. Microscopy was used to identify leukocytes counts in urine which was reported per high power field. Only a count equal to or greater than five leucocytes per high power field was considered significant.

#### Urine culture

At least 5ul of the urine sample was pipetted with sterile micropipette tip, dispensed in the middle of a sterile medium and spread with a spreader and incubated the plates at 37 °C for 24 h. CLED and MacConkey media were used for isolation of gram-negative bacteria mainly coliforms and Enterobacteriaceae. Chocolate and blood agar were used for cultivation of gram-positive bacteria. CFU were manually counted by marking with a fine permanent marker. Colony counts yielding bacterial growth of 1000 (10^3^) with significant pyuria per ml of urine sample was taken as a significantly positive culture. No significant growth meant bacterial growth below threshold of 10^3^, and mixed growth meant growth of more than 2 organisms which were taken as contaminants. Identification of bacteria were done using colony characteristics, gram stain reaction of the organisms and biochemical tests which included but not limited to catalase and coagulase tests for Staphylococcus species, Indole, citrate, motility, urease*,* starch hydrolysis, lactose utilization, casein hydrolysis and hydrogen sulphide gas production tests for Enterobacteriaceae. Urine culture was performed before microscopy to avoid contamination. Susceptibility testing was done on all significant growth using Mueller Hinton Agar following standard criteria following the Kirby Bauer method [11, 12]. Suspensions of standard *Escherichia coli* ATCC25922 (for gram-negative) and *Staphylococcus aureus ATCC25923* (for gram- positive) were used. The zones of clearance (diameters) around each disc were measured in millimeters using a ruler, and compared against the zone diameter of the standard organism as recommended by the Clinical and Laboratory Standards Institute - USA. In addition, *Escherichia coli* and *Klebsiella pneumoniae* were screened for ESBL production by the phenotypic method and those positive were confirmed by the phenotypic confirmatory test as per Clinical and Laboratory Standards Institute (CLSI) guidelines)*.* For detection of MRSA, Oxacillin 6 mg/L disc were used on a Mueller-Hinton agar. All participants were treated empirically with Nitrofurantoin (100 mg/TID) until definitive Culture results were obtained and utilized to guide therapy.

Data was cleaned, coded and entered in RED-cap version 8.2 and a back -up made. It was then exported to STATA 14.1 for analysis. The baseline characteristics of participants were summarized using frequencies, mean with Standard Deviation (SD) and median (with IQR). The proportion of participants with positive urine cultures was expressed as a percentage of the total study participants. Isolated and identified uropathogens were each quantified as percentage of total isolated organisms. The susceptibility patterns of isolated aetiologic organisms to commonly prescribed antibiotics were expressed as frequencies and percentages. Isolates of ESBL and MRSA were expressed as percentages of the total bacterial pathogens isolated. Used binary logistic regression to determine the independent effect of the variables (age, frequency of coitus, gravidity, gest age, prior UTI diagnosis and chronic illness) by calculating the strength of the association between UTI and associated factors using odds ratio (OR) and 95% confidence interval (CI). Adjusted OR (for variables which were statistically significant in binary logistic analysis) was computed using multivariable logistic regression to control the confounding variables. A *p*-value of < 0.05 was considered as an indicator of statistical significance. Ethical approval was granted by Mbarara University of Science and Technology Research ethics committee (reference number 11/09–19) and administrative permission was received from the Director of Mbarara Regional Referral approval. A written informed consent was sought for and received from each participant prior to study enrolment. For each culture-positive result, the clinician was contacted and treatment was given as per the result and antibiotic susceptibility pattern.

## Results

We enrolled a total of 400 pregnant women with symptomatic urinary tract infection. The mean age of the participants was 27.1 (±5.3) years,), living with a partner (94.25%), were in informal employment (76.25%) and had attained at least secondary level education (65.75%) (Tables 1 and 2).

**Table 1:** Socio-demographic characteristics of study participants by diagnosis of UTI

	Overall (*N* = 400)	Positive Culture (*N* = 140)	Negative Culture (*N* = 260)	
Characteristic	n/N (%)	n/N (%)	n/N (%)	*p*-value
Age in years, mean (±SD)	27.1 (±5.3)	27.7 (±5.5)	26.7 (±5.1)	0.075
Education level				0.543
None	6 (1.50)	1 (0.71)	5 (1.92)	
Primary	131 (32.75)	48 (34.29)	83 (31.92)	
Secondary	167 (41.75)	62 (44.29)	105 (40.38)	
Tertiary	96 (24.00)	29 (20.71)	67 (25.77)	
Occupation				0.531
Informal Employment	307 (76.25)	140 (77.86)	198 (76.15)	
Employment	93 (23.25)	31 (22.14)	62 (23.85)	
Marital status				0.115
Does not leave with partner	23 (5.75)	6 (4.29)	17 (6.54)	
Lives with a partner	377 (94.25)	134 (95.71)	243 (93.46)	
Frequency of coitus per week, median (IQR)	2 (1 4)	3 (1 4)	2 (1 3)	0.166
Cleaning of genitalia				0.903
Back to front	267 (66.75)	94 (67.14)	173 (66.54)	
Front to back	133 (33.25)	46 (32.86)	87 (33.46)

**Table 2:** Medical and Obstetric factors of the study participants

	Overall (*N* = 400)	Positive Culture (*N* = 140)	Negative Culture(*N* = 260)	
Characteristic	n/N (%)	n/N (%)	n/N (%)	*p* value
Gravidity				0.301
Prime-gravid	114 (28.43)	39 (27.86)	75 (28.85)	
Multi-gravid	218 (54.61)	71 (50.71)	147 (56.54)	
Grand-multi-gravid	68 (16.96)	30 (21.43)	38 (14.62)	
Trimesters				0.371
1st	65 (16.25)	19 (13.57)	46 (17.70)	
2nd	149 (37.25)	55 (39.29)	94 (36.15)	
3rd	186 (46.50)	66 (47.14)	120 (46.15)	
Ever diagnosed with UTI before in this pregnancy	179 (44.75)	81 (57.86)	98 (37.69)	< 0.001
Had symptomatic UTI before in this pregnancy	138 (34.50)	51 (36.43)	87 (33.46)	0.552
Got treatment for UTI symptoms	92 (66.67)	35 (38.00)	57 (61.96)	0.708
Symptomatology of UTI				
Painful micturition	219 (55.16)	81 (58.27)	138 (53.49)	0.360
Increased frequency	235 (59.19)	86 (61.43)	149 (57.98)	0.504
Urgency of passing urine	168 (42.21)	59 (42.45)	109 (42.08)	0.945
Urine color change	247 (61.75)	89 (63.57)	158 60.77)	0.582
Lower abdominal pain	389 (97.25)	35 (96.43)	254 (97.69)	0.461
Fever	80 (20.10)	30 (21.74)	50 (19.23)	0.552
Vomiting	90 (22.50)	27 (19.29)	63 (24.23)	0.259
Amount of urine change				0.639
Same	85 (21.30)	30 (21.58)	55 (21.15)	
Reduced	217 (54.39)	79 (56.83)	138 (53.08)	
Increased	97 (24.31)	30 (21.58)	67 (25.77)	
Abnormal vaginal discharge, yes	214 (53.63)	78 (55.71)	136 (52.51)	0.54
Chronic conditions present				0.024
Diabetes	6 (1.50)	4 (2.86)	2 (0.77)	
Hypertension	6 (1.50)	1 (0.71)	5 (1.92)	
HIV/AIDS	30 (7.50)	17 (12.14)	13 (5.00)	
Other illness	3 (0.75)	1 (0.71)	2 (0.77)	
None	355 (88.75)	117 (83.57)	238 (91.54)	

The proportion of culture-positive UTI was 35% (140/400). Six different bacteria were isolated in this study with majority of the isolates 107 (76.43%) being Gram-negative *Klebsiella pneumoniae* was found to be the most frequent Gram-negative isolate (37.41%), followed by *E. coli* (28.78%), *Pseudomonas aeruginosa* and *Proteus mirabilis* (each 5.04%) and Citrobacter freundii (~ 1%). *Staphylococcus aureus* was the only Gram-positive bacteria isolated (23.57%). Generally, organisms were sensitive to Gentamicin, Ceftriaxone, Imipenem, Cefotaxime, Ciprofloxacin, Nitrofurantoin and Cefuroxime (79.3, 82.9, 85.0, 81.4, 78.6, 66.4 and 65.7% respectively). Organisms were resistant to Ampicillin, Amoxicillin and Amoxicillin/Clavulanic acid (95.0, 95.7 and 72.9% respectively). ESBL-producing organisms were 29.0 and 33.3% of the *Staphylococcus aureus* were MRSA. All cultures demonstrated resistance to more than one drug (Tables 3, 4 and 5).

**Table 3:** Laboratory findings

Test	Result	Frequency (n)	Percent (%)
Microscopy (pyuria)	Pus cells present	166	41.60
	No pus cells	234	58.40
Significant pus cells			
	Yes (WBCs> 5)	157	94.58
	No	9	5.42
Urine Culture results (*N* = 400)			
	Significant growth	140	35.00
	Non-significant growth	250	62.50
	Mixed growth	10	2.50
Gram test (*N* = 140)			
	Positive	33	23.57
	Negative	107	76.43
Microorganisms (*N* = 140)			
	Citrobacter freundii	1	0.72
	*E. coli*	40	28.78
	*Klebsiella pneumoniae*	52	37.41
	*Proteus mirabilis*	7	5.04
	Pseudomonas	7	5.04
	*Staphylococcus aureus*	33	23.57
ESBL (*N* = 107)			
	Yes	31	29.0
	No	76	71.0
MRSA (*N* = 33)			
	Yes	11	33.3
	No	22	66.7

**Table 4:** Antimicrobial susceptibility patterns of bacteria isolated from urine samples

	Susceptibility	Bacterial Isolates						
Antibiotics		*Klebsiella*	*E. coli*	*Proteus*	*Pseudomonas*	*Citrobacter*	*S. aureus*	Total
AMP	S	1 (1.9)	2 (5.0)	1 (14.3)	0 (0.0)	0 (0.0)	3 (9.1)	7 (5.0)
	R	51 (98.1)	38 (95.0)	6 (85.7)	7 (100.0)	1 (100.0)	30 (90.1)	133 (95.0)
AMO	S	2 (3.8)	0 (0.0)	2 (28.6)	0 (0.0)	0 (0.0)	2 (6.1)	6 (4.3)
	R	50 (96.2)	40 (100.0)	5 (71.4)	7 (100.0)	1 (100.0)	31 (93.9)	134 (95.7)
AMOCLAV	S	13 (25.0)	13 (32.5)	4 (57.1)	1 (14.3)	0 (0.0)	7 (21.2)	38 (27.1)
	R	39 (75.0)	27 (67.5)	3 (42.9)	6 (85.7)	1 (100.0)	26 (78.8)	102 (72.9)
NITRO	S	27 (51.9)	32 (80.0)	6 (85.7)	0 (0.0)	1 (100.0)	27 (81.8)	93 (66.4)
	R	25 (48.1)	8 (20.0)	1 (14.3)	7 (100.0)	0 (0.0)	6 (18.2)	47 (33.6)
CAF	S	34 (65.4)	23 (57.5)	3 (42.9)	5 (71.4)	0 (0.0)	17 (51.5)	82 (58.6)
	R	18 (34.6)	17 (42.5)	4 (57.1)	2 (28.6)	1 (100.0)	16 (48.5)	58 (41.4)
CIPRO	S	47 (90.4)	31 (77.5)	6 (85.7)	3 (42.9)	1 (100.0)	22 (66.7)	110 (78.6)
	R	5 (9.6)	9 (22.5)	1 (14.3)	4 (57.1)	0 (0.0)	11 (33.3)	30 (21.4)
GENT	S	47 (90.4)	31 (77.5)	6 (85.7)	3 (42.9)	1 (100.0)	23 (69.7)	111 (79.3)
	R	5 (9.6)	9 (22.5)	1 (14.3)	4 (57.1)	0 (0.0)	10 (30.3)	29 (20.7)
CEFTCLAV	S	31 (59.6)	22 (55.0)	4 (57.1)	4 (57.1)	0 (0.0)	8 (24.2)	69 (49.3)
	R	21 (40.4)	18 (45.0)	3 (42.9)	3 (42.9)	1 (100.0)	25 (75.8)	71 (50.7)
CEFOTAX	S	44 (84.6)	38 (95.0)	7 (100.0)	6 (85.7)	1 (100.0)	18 (54.5)	114 (81.4)
	R	8 (15.4)	2 (5.0)	0 (0.0)	1 (14.3)	0 (0.0)	15 (45.5)	26 (18.6)
CEFUROX	S	32 (61.5)	28 (70.0)	5 (71.4)	4 (57.1)	1 (100.0)	22 (66.7)	92 (65.7)
	R	20 (38.5)	12 (30.0)	2 (28.6)	3 (42.9)	0 (0.0)	11 (33.3)	48 (34.4)
CEFTRI	S	47 (90.4)	33 (83.3)	7 (100.0)	7 (100.0)	1 (100.0)	21 (63.6)	116 (82.9)
	R	5 (9.6)	7 (16.7)	0 (0.0)	0 (0.0)	0 (0.0)	12 (36.4)	24 (17.4)
IMIPEN	S	50 (96.2)	33 (83.3)	5 (71.4)	4 (57.1)	1 (100.0)	26 (78.8)	119 (85.0)
	R	2 (3.8)	7 (16.7)	2 (28.6)	3 (42.9)	0 (0.0)	7 (21.2)	21 (15.0)
ERYTH	S						18 (54.5)	18 (54.5)
	R						15 (45.5)	15 (45.5)
METH	S						22 (66.7)	22 (66.7)
	R						11 (33.3)	11 (33.3)

*AMP* Ampicillin, *AMO* Amoxicillin, *AMOCLAV* Amoxicillin/Clavulanic acid, *NITRO* Nitrofurantoin, *CAF* Chloramphenicol, *CIPRO* Ciprofloxacin, *GENT* Gentamicin, *CEFTCLAV* Ceftazidime/Clavulanic acid, *CEFOTAX* Cefotaxime, *CEFUROX* Cefuroxime, *CEFTRI* Ceftriaxone, *IMIPEN* Imipenem, *ERYTH* Erythromycin, METH Methicillin, *S. aureus* Staphylococcus aureus, *E. coli Escherichia coli*, *S* Sensitive, *R* Resistant

**Table 5:** Bivariate and multivariate logistic regression analyses for factors associated with symptomatic UTI

	% Culture-positive UTI	Bivariate Analysis		Multivariate Analysis	
Characteristic	n/N (%)	OR (95%CI)	***p*** value	Adjusted OR (95%CI)	***p*** value
**Age category in years**					
< 25 yrs.	53/180 (29.44)	Ref		Ref	
25 yrs. & above	87/220 (39.55)	1.57 (1.03–2.38)	0.036	2.16 (0.93–2.20)	0.102
**Frequency of coitus**					
None	25/85 (29.41)	Ref			
1–5 times/week	108/297 (36.36)	1.37 (0.81–2.31)	0.237		
> 5 times/week	7/18 (38.89)	1.53 (0.53–4.39)	0.432		
**Gravidity**					
Prime gravida	39/114 (34.21)	Ref			
2–4 pregnancies	71/218 (32.57)	0.93 (0.57–1.50)	0.763		
5 & above pregnancies	30/67 (44.78)	1.56 (0.84–2.89)	0.159		
**Gestational age**					
1st Trimester	37/103 (35.92)	Ref			
2nd Trimester	76/207 (36.71)	1.03 (0.63–1.69)	0.891		
3rd Trimester & above	27/89 (30.34)	0.78 (0.42–1.42)	0.413		
**Diagnosed with UTI before**					
No	59/221 (26.70)	Ref		Ref	
Yes	81/179 (45.25)	2.27 (1.49–3.45)	< 0.001	1.98 (1.28–3.05)	0.002
**Chronic illnesses**					
None	117/355 (32.96)	Ref		Ref	
DM	4/6 (66.67)	4.07 (0.73–22.53)	0.108	2.70 (0.48–15.29)	0.261
HIV	17/30 (56.67)	2.66 (1.25–5.66)	0.011	2.07 (0.95–4.50)	0.067
Other illnesses	2/9 (22.22)	0.58 (0.12–2.84)	0.503	0.72 (0.15–3.58)	0.690

*Ref* Reference category, *OR Odds ratio*, *CI* Confidence interval*, DM* Diabetes mellitus

*HIV* Human Immuno-deficiency Virus*, AIDS* Acquired Immuno-deficiency Syndrome

One factor was significantly associated with a culture-positive UTI which is a history of a previous diagnosis of UTI. All the other factors were not statistically significant.

## Discussion

The prevalence of UTI in this study was 35%. This was slightly higher than the global prevalence that ranges from 13 to 33%. It however was comparable to the prevalence reported in the study done in Bale Zone, South-east Ethiopia by Solomon Taye et al. in which the prevalence was reported to be 35.3%. This was much higher than the prevalence reported in other studies; A study in Ethiopia by Kedebe et al. in 2016 found a prevalence of 11.5%, in Eastern Tanzania by Matalingana in 2015 it was at 16.1%, at Pumwani Maternity Hospital, Nairobi, Kenya by Onyango et al. in 2018 it was 15.7% and at Mulago National Referral Hospital by Wanyama in 2003 the prevalence of symptomatic bacteriuria was at 15%. The prevalence of culture-proven UTI among pregnant women with UTI symptoms was 4% in another Mulago based study by Musa Sekikubo in 2017. All these studies had smaller sample sizes than our study. Also, the variation may have been due to differences in the environmental conditions, social habits in the community, the standard of personal hygiene and health care seeking habits.

Similar to other studies in pregnant women with symptomatic UTI, Bale Zone, Southeast Ethiopia by Solomon Taye et al. in 2018, Francois de Paul Siemefo Kamgang et al. in Durban, South Africa in 2016, a meta-analysis by Feizollah Mansouri et al., Onyango et al. in 2018 at Pumwani Maternity Hospital, in Kenya and Andabati G and Byamugisha J in 2010 at Mulago the dominant pathogenic agents were Gram-negative bacteria. However, unlike previous authors who had reported E. coli as the commonest pathogen, Klebsiella pneumoniae was the most dominant followed by E. coli in this study. Traditionally, E. coli has been the dominant uropathogen owing to its possession of toxins, adhesins, pili and fimbriae that allow adherence to uroepithelium. These protect the bacteria from urinary clearance and allow bacterial multiplication and uroepithelial tissue invasion. Recent studies however indicate that Klebsiella pneumoniae which has traditionally been a nosocomial organism is an emerging dominant community acquired uropathogen [3, 6–9]. This has been attributed to its emerging and inherent virulent factors that include capsule, lipopolysaccharide, Siderophore, types 1 and 3 fimbriae, biofilm formation, and antibiotic resistance. Other Gram-negative organisms isolated in our study were Proteus mirabilis, *Pseudomonas aeruginosa* and Citrobacter freundii similar to other studies [13]. Staphylococcus aureus was the only Gram-positive isolate in our study. This is similar to the study done by Okonkwo in Ibadan, South-Western Nigeria in 2009, Adelaide Oguti et al. in Kenya, Deus Kabugo et al. at Mulago National hospital who reported Staphylococcus aureus as the most common gram-positive uropathogen in pregnant women, unlike in other studies which isolated coagulase negative staphylococcus and enterococcus as the most dominant Gram-positive uropathogens. This was worrying owing to its potential of causing severe infections to the mother and the new born [13–15].

Generally, all organisms were highly resistant to Amoxicillin, Ampicillin, Amoxicillin/Clavulanic acid and Ceftazidime/Clavulanic acid at 95.7, 95.0,72.9 and 50.7% respectively yet these are the commonly prescribed antibiotics, in the study setting. Klebsiella pneumonia, the dominant isolate in our study was 98.1 and 96% resistant to Ampicillin and Amoxicillin respectively. E. coli and *Pseudomonas aeruginosa* were 100% resistant to both Ampicillin and Amoxicillin. This has been demonstrated in other studies in Ethiopia, Kenya, Tanzania and Uganda at Mulago National Referral Hospital though the level of resistance was much higher than reported in most of the mentioned studies [8, 10, 13, 16, 17]. The high resistance could be attributed to the over-the counter availability of these drugs particularly Ampicillin and Amoxicillin. All isolates were sensitive to Ceftriaxone, Cefotaxime, Gentamicin, Ciprofloxacin, Nitrofurantoin and Cefuroxime at 82.9, 81.4, and 79.3%, 78.6, 66.4 and 65.7% respectively. Klebsiella pneumoniae was particularly highly sensitive to Gentamicin, Ceftriaxone, Ciprofloxacin and Cefotaxime. E. coli and Proteus were particularly highly sensitive to Nitrofurantoin, Ciprofloxacin, Gentamicin, Cefotaxime and Ceftriaxone. Pseudomonas was sensitive only to Cefotaxime and Ceftriaxone and also showed a 43% resistance to Imipenem. The sensitivity patterns exhibited by the Gram-negative isolates in our study have been demonstrated in other studies in Ethiopia, Nigeria, Uganda, Kenya, Cameroon and South Africa [8–10, 13, 16, 17]. The prevalence of MRSA was 33.3% in our study which was higher than that found by Onyango et al. at Puwani Hospital in Nairobi in 2018 (20%) [18]. The presence of ESBL producers among the Enterobacteriaceae in our study was 29.0% with Klebsiella at 25% and E. coli at 32.5% respectively. This was higher than that by Onyango et al. 2018 in Kenya (19%), Musa Sekikubo et al. 2017 at Mulago (18%) but was less than that found by Sabrina et al. 2010 in Tanzania (32.5%) [10, 18, 19]. A meta-analysis by Feizollah Mansouri et al. 2019 found the pooled prevalence of ESBL-producing Enterobacteriaceae of 25% with 45% in Africa, 33% in India, 5% in Europe and 3% in North America. Presence of multi-drug resistant (MDR) organisms (resistance to ≥2 drugs) was 100% which was the same as was found by Behailu Derese et al. 2016 which was much higher than that found by Sabrina et al. 2010 in Tanzania (77%), Sekikubo et al. 2017 at Mulago (36%), Taye et al. 2018 in Ethiopia (only E.coli was MDR) [10, 18, 19].

A history of a previous diagnosis of UTI was significantly associated with culture positive UTI. Previous diagnosis of UTI was also reported by Behailu Derese et al. 2016 in Eastern Ethiopia to be significantly associated with UTI in pregnancy. This might be due to the presence of resistance strains from those who had the previous history of UTI [20].

## Conclusion

Our study recorded a higher prevalence of culture-positive UTI in pregnancy than most of the studies in East Africa and Ethiopia. The dominant isolates in our study were Klebsiella pneumoniae and E. coli. These two organisms were highly resistant to the commonly used antibiotics. Prevalence of ESBL-producing Enterobacteriaceae and MRSA was high in our study.

UTI in pregnancy was significantly associated with previous UTI. We encourage all clinicians to base diagnosis of UTI in pregnancy on urine culture. Empirical treatment of UTI should be avoided as sensitivity varies for each organism, for each drug and over time. Particular interest should be given to pregnant women with a history of UTI. We should also educate pregnant women to avoid over-the-counter antibiotics as this is likely to worsen antibiotic resistance. Also, we recommend screening for bacteriuria in all pregnant women.

## Supplementary Information

**Additional file 1.**

## Data Availability

The datasets used and/or analyzed during the current study are available from the corresponding author on reasonable request.

## Abbreviations

CFU: Colony Forming Unit
CLED: Cysteine–lactose–electrolyte-deficient agar or medium
CLSI: Clinical and Laboratory Standards Institute
ESBL: Extended spectrum ß-Lactamase
IQR: Interquartile range
MRRH: Mbarara Regional Referral Hospital
MRSA: Methicillin Resistant *Staphylococcus aureus*
MSU: Midstream urine
RED-cap: Research Electronic Data capture
SD: Standard Deviation
UTI: Urinary Tract Infection
MDR: Multidrug resistant
OR: Odds ratio
HIV: Human Immuno-deficiency Virus
AIDS: Acquired Immuno-deficiency Syndrome
*E. coli*: *Escherichia coli*
*K. pneumoniae*: *Klebsiella pneumoniae*
