# Supplementary material for: Prevalence and bacteriology of culture-positive urinary tract infection among pregnant women with suspected urinary tract infection at Mbarara regional referral hospital, South-Western Uganda
Source: BMC Pregnancy Childbirth. 2021 Feb 23;21:159. doi: 10.1186/s12884-021-03641-8 (PMC7903640; doi:10.1186/s12884-021-03641-8)
Supplement: Supplementary file 1 — Additional file 1. [file 12884_2021_3641_MOESM1_ESM.pdf]

A) Demographic characteristics

Record ID	________________
Antenatal number	________________
Serial number	________________
Date of interview	________________
Initials	________________
Address	☐ Village<br>☐ Sub-county<br>☐ District
Village	________________
Sub-county	________________
District	________________

B) Social History

Age in years completed: __________________________________

Level of Education

- ◯ None
- ◯ Primary
- ◯ Secondary
- ◯ Tertiary

C) Occupation

Occupation	◯ Un employed/ full time house wife<br>◯ Skilled employment<br>◯ Peasant farmer<br>◯ Student<br>◯ Others
Other occupation	__________________________________

D) Marital Status

Marital Status	◯ Single<br>◯ Married / cohabiting<br>◯ Separated<br>◯ Divorced<br>◯ Widowed

E) Religious Affiliation / faith

Religious Affiliation / faith	◯ Roman Catholic<br>◯ Protestant Church of Uganda<br>◯ Muslims<br>◯ Others
Other Religion	__________________________
F) Frequency of sexual intercourse during current pregnancy, number of times in a week:	__________________________

G) How do you clear your genitalia

How do you clear your genitalia	◯ Cleans genitals from back to front<br>◯ Cleans genitals from front to back

H) Obstetric History

Obstetric History	☐ a) Gravidity<br>☐ b) Parity<br>☐ c) Last normal menstruation period: (date/ Month/year)<br>☐ d) Weeks of amenorrhea
Gravidity	__________________________________
Parity	__________________________________
LNMP	__________________________________
Weeks of amenorrhea	__________________________________

I) Antenatal visit

Antenatal visit	◯ 1st visit<br>◯ 2nd visits<br>◯ 3rd visit<br>◯ 4th visit<br>◯ others specify
Other Antenatal visits	__________________________________

J)Previous history of UTI

Have you ever been told that you're suffering from UTI before this pregnancy?	◯ Yes<br>◯ No
Did you have any of the following symptoms before this pregnancy, frequency of micturition, pain on passing urine, lower abdominal pain, loin pain	◯ Yes<br>◯ No

K) Complication encountered in this pregnancy

About micturition (in the last 2 weeks)	______________________
Painful / burning	◯ Yes<br>◯ No
Increased frequency	◯ Yes<br>◯ No
Urgency of passing urine	◯ Yes<br>◯ No
Urine has changed color	◯ Yes<br>◯ No
If yes, what is the color of urine	◯ Dark<br>◯ Yellow<br>◯ Reddish<br>◯ Whitish<br>◯ Others specify
Other color of urine	______________________
Amount of urine passed since you became sick	◯ Same<br>◯ Reduced<br>◯ Increased
Any history of abnormal vaginal discharge	◯ Yes<br>◯ No
What is the color of the discharge	◯ Pus-like<br>◯ Milk-like<br>◯ Blood like<br>◯ 0thers (specify
History of lower abdominal pain	◯ Yes<br>◯ No
Fever	◯ Yes<br>◯ No
Vomiting	◯ Yes<br>◯ No
If yes to any of the above symptoms, have you suffered from similar illness before in this pregnancy?	◯ Yes<br>◯ No
If yes did you get treatment for it?	◯ Yes<br>◯ No

If yes what kind of treatment did you get (Name of drugs and number of times drugs was taken in a day & duration of treatment?	☐ Tablets<br>☐ Injection<br>☐ Capsules<br>☐ All above
How long ago did you stop treatment?	☐ 7 days ago<br>☐ 7-14 days ago<br>☐ 14-21 days ago
Did you have similar conditions before in this current pregnancy?	○ Yes<br>○ No
Any history of instrumentation (Catheter or speculum) in this current pregnant?	○ Yes<br>○ No
Chronic conditions present?	○ Diabetes<br>○ Sickle cell disease<br>○ Hypertension<br>○ HIV/AIDS<br>○ Others specify<br>○ None of the above
Other chronic conditions	__________________________________
Contraceptive methods ever used and when?	○ Spermicidal cream/jelly<br>○ Diagpharagm/cap<br>○ Injectaplan/Depo provera<br>○ Norplant<br>○ IUCD<br>○ Oral contraceptives<br>○ Non

Laboratory form

Laboratory results for urine Date	__________________________________
Patient serial NO	__________________________________
Microscopy	◯ Yes<br>◯ No
If yes	◯ Pus cells<br>◯ No pus cells
Significant pus cell	◯ Yes (number of WBCS>5)<br>◯ No
Bacterial in results	◯ Yes<br>◯ No
Culture results	◯ Significant growth<br>◯ No significant growth<br>◯ Mixed growth
Gram test	◯ Positive<br>◯ Negative
1, Micro organism	__________________________________

G) Susceptibility

	Sensitive	Resistant
Amoxacillin	☐	☐
Amoxiclav	☐	☐
Ampicillin	☐	☐
Gentmicin	☐	☐
Erythromicin	☐	☐
Nitrofurantoin	☐	☐
Metronidazole	☐	☐
Chloramphenicol	☐	☐
Ciprofoxacin	☐	☐
Ceftazidimeclav	☐	☐
Cefuroxime	☐	☐
Cefotaxime	☐	☐
Cetriaxone	☐	☐

Imipenem	☐	☐
Methicillin	☐	☐

If gram Negative, is it ESBL?
- ◯ Yes
- ◯ No

If S.aureus, is it MRSA?
- ◯ Yes
- ◯ No
